# 4-(2,7-Dimethyl-4-oxo-1,3-thia­zolo[4,5-*d*]pyridazin-5-yl)benzene­sulfonamide

**DOI:** 10.1107/S1600536811021271

**Published:** 2011-06-18

**Authors:** Abdullah M. Asiri, Hassan M. Faidallah, Seik Weng Ng

**Affiliations:** a Chemistry Department, Faculty of Science, King Abdul Aziz University, Jeddah 21589, Saudi Arabia; b Department of Chemistry, University of Malaya, 50603 Kuala Lumpur, Malaysia

## Abstract

The thia­zole–pyridazine fused-ring system of the title compound, C_13_H_12_N_4_O_3_S_2_, is approximately planar (r.m.s. deviation = 0.037 Å); the benzene ring connected to the fused-ring system through the N atom is twisted by 39.3 (1)°. The amine group uses an H atom to form a hydrogen bond to the ketonic O atom of an inversion-related mol­ecule to generate a dimer; adjacent dimers are linked by an N—H⋯O hydrogen bond to form a linear chain.

## Related literature

For background to related compounds, see: Makki & Faidallah (1996 ▶).
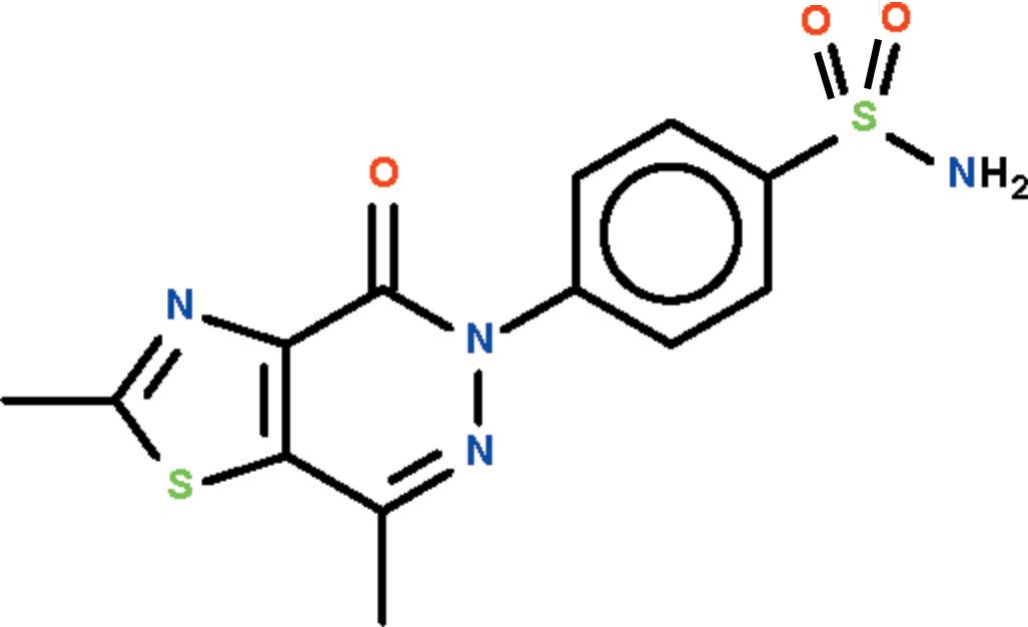


## Experimental

### 

#### Crystal data

C_13_H_12_N_4_O_3_S_2_
*M* *_r_* = 336.39
Monoclinic, 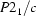

*a* = 12.6048 (10) Å
*b* = 13.2273 (10) Å
*c* = 8.9703 (7) Å
β = 102.242 (1)°
*V* = 1461.6 (2) Å^3^
*Z* = 4
Mo *K*α radiation
μ = 0.38 mm^−1^
*T* = 100 K
0.20 × 0.15 × 0.15 mm

#### Data collection

Bruker SMART APEX diffractometer
Absorption correction: multi-scan (*SADABS*; Sheldrick, 1996 ▶) *T*_min_ = 0.928, *T*_max_ = 0.945
9962 measured reflections
3333 independent reflections
2888 reflections with *I* > 2σ(*I*)
*R*_int_ = 0.028

#### Refinement

*R*[*F* ^2^ > 2σ(*F* ^2^)] = 0.034
*wR*(*F* ^2^) = 0.097
*S* = 1.06
3333 reflections
209 parameters
2 restraints
H atoms treated by a mixture of independent and constrained refinement
Δρ_max_ = 0.39 e Å^−3^
Δρ_min_ = −0.53 e Å^−3^

### 

Data collection: *APEX2* (Bruker, 2009 ▶); cell refinement: *SAINT* (Bruker, 2009 ▶); data reduction: *SAINT*; program(s) used to solve structure: *SHELXS97* (Sheldrick, 2008 ▶); program(s) used to refine structure: *SHELXL97* (Sheldrick, 2008 ▶); molecular graphics: *X-SEED* (Barbour, 2001 ▶); software used to prepare material for publication: *publCIF* (Westrip, 2010 ▶).

## Supplementary Material

Crystal structure: contains datablock(s) global, I. DOI: 10.1107/S1600536811021271/xu5226sup1.cif

Structure factors: contains datablock(s) I. DOI: 10.1107/S1600536811021271/xu5226Isup2.hkl

Supplementary material file. DOI: 10.1107/S1600536811021271/xu5226Isup3.cml

Additional supplementary materials:  crystallographic information; 3D view; checkCIF report

## Figures and Tables

**Table 1:** Hydrogen-bond geometry (Å, °)

*D*—H⋯*A*	*D*—H	H⋯*A*	*D*⋯*A*	*D*—H⋯*A*
N4—H1⋯O1^i^	0.87 (1)	2.06 (1)	2.922 (2)	169 (2)
N4—H2⋯O2^ii^	0.88 (1)	2.38 (2)	3.090 (2)	139 (2)

Symmetry codes: (i) 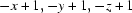; (ii) 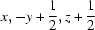.

**Fig. 1.**
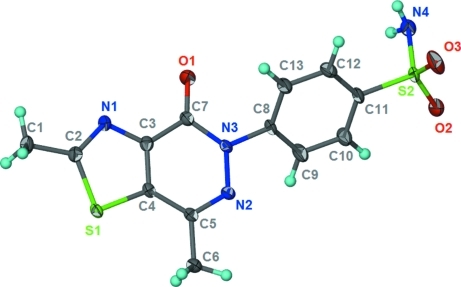
Thermal ellipsoid plot (Barbour, 2001) of C13H12N4O3S2 at the 70% probability level; hydrogen atoms are drawn as spheres of arbitrary radius.

**Fig. 2.**
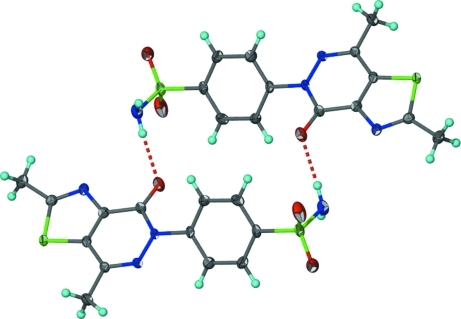
Hydrogen-bonded dimeric structure.

## supplementary crystallographic information

### Comment

This compound belongs to a class of tricyclic compounds posessing high antibacterial activity that are synthesized by reacting an aryl hydrazine with a thiazole that bears acetyl and carboxyl substituents on adjacent carbon atoms (Makki & Faidallah, 1996). A sulfonamido unit in the benzene ring of phenyl hydrazine should improved the activity. The thiazole–pyridazine fused-ring of C_13_H_12_N_4_O_3_S_2_ (Scheme I, Fig. 1) is planar; the benzene ring that bears the sulfonamido unit is twisted by 39.3 (1)°. The amino group uses an H atom to form a hydrogen bond to the ketonic O atom of an inversion-related molecule to generate a dimer (Fig. 2); adjacent dimers are linked by a weaker N–H···O hydrogen bond to form a linear chain (Table 1).

### Experimental

Ethyl 5-acetyl-2-methylthiazole-4-carboxylate (0.40 g, 0.002 mol) in ethanol (25 ml) was heated with *p*-sulfonamidophenyl hydrazine hydrochloride (0.49 g, 0.002 mol) for 2 h. The pyridazine that separated was collected and recrystallized from ethanol.

### Refinement

Carbon-bound H-atoms were placed in calculated positions (C–H 0.95 to 0.98 Å) and were included in the refinement in the riding model approximation, with *U*(H) set to 1.2 to 1.5*U*_eq_(C). The amino H-atoms were located in a difference Fourier map, and were refined with a distance restraint of N–H 0.88±0.01 Å; temperature factors were refined. Omitted because of bad disagreement were (12 2 3), (1 0 0) and (-1 2 1).

### Crystal data

**Table:**

C_13_H_12_N_4_O_3_S_2_	*F*(000) = 696
*M**_r_* = 336.39	*D*_x_ = 1.529 Mg m^−^^3^
Monoclinic, *P*2_1_/*c*	Mo *K*α radiation, λ = 0.71073 Å
Hall symbol: -P 2ybc	Cell parameters from 3931 reflections
*a* = 12.6048 (10) Å	θ = 2.3–28.2°
*b* = 13.2273 (10) Å	µ = 0.38 mm^−^^1^
*c* = 8.9703 (7) Å	*T* = 100 K
β = 102.242 (1)°	Block, light brown
*V* = 1461.6 (2) Å^3^	0.20 × 0.15 × 0.15 mm
*Z* = 4	

### Data collection

**Table:**

Bruker SMART APEX diffractometer	3333 independent reflections
Radiation source: fine-focus sealed tube	2888 reflections with *I* > 2σ(*I*)
graphite	*R*_int_ = 0.028
ω scans	θ_max_ = 27.5°, θ_min_ = 2.3°
Absorption correction: multi-scan (*SADABS*; Sheldrick, 1996)	*h* = −11→16
*T*_min_ = 0.928, *T*_max_ = 0.945	*k* = −17→17
9962 measured reflections	*l* = −11→11

### Refinement

**Table:**

Refinement on *F*^2^	Primary atom site location: structure-invariant direct methods
Least-squares matrix: full	Secondary atom site location: difference Fourier map
*R*[*F*^2^ > 2σ(*F*^2^)] = 0.034	Hydrogen site location: inferred from neighbouring sites
*wR*(*F*^2^) = 0.097	H atoms treated by a mixture of independent and constrained refinement
*S* = 1.06	*w* = 1/[σ^2^(*F*_o_^2^) + (0.0449*P*)^2^ + 0.9855*P*] where *P* = (*F*_o_^2^ + 2*F*_c_^2^)/3
3333 reflections	(Δ/σ)_max_ = 0.001
209 parameters	Δρ_max_ = 0.39 e Å^−^^3^
2 restraints	Δρ_min_ = −0.53 e Å^−^^3^

### Special details

**Table:**

Geometry. All e.s.d.'s (except the e.s.d. in the dihedral angle between two l.s. planes) are estimated using the full covariance matrix. The cell e.s.d.'s are taken into account individually in the estimation of e.s.d.'s in distances, angles and torsion angles; correlations between e.s.d.'s in cell parameters are only used when they are defined by crystal symmetry. An approximate (isotropic) treatment of cell e.s.d.'s is used for estimating e.s.d.'s involving l.s. planes.

### Fractional atomic coordinates and isotropic or equivalent isotropic displacement parameters (Å^2^)

**Table:**

	*x*	*y*	*z*	*U*_iso_*/*U*_eq_	
S1	1.13140 (3)	0.42793 (3)	0.70269 (5)	0.01393 (12)	
S2	0.34576 (3)	0.29976 (4)	0.11537 (5)	0.01750 (12)	
O1	0.73961 (10)	0.47213 (10)	0.67068 (14)	0.0220 (3)	
O2	0.33900 (11)	0.19520 (11)	0.07281 (17)	0.0305 (3)	
O3	0.32382 (11)	0.37373 (12)	−0.00328 (15)	0.0302 (3)	
C1	1.14993 (15)	0.57418 (14)	0.9320 (2)	0.0203 (4)	
H1A	1.1070	0.6150	0.9888	0.030*	
H1B	1.1924	0.6189	0.8799	0.030*	
H1C	1.1991	0.5302	1.0029	0.030*	
C2	1.07602 (14)	0.51116 (13)	0.81743 (19)	0.0155 (3)	
N1	0.97011 (12)	0.51401 (11)	0.79149 (16)	0.0160 (3)	
N2	0.86442 (11)	0.31764 (11)	0.42826 (16)	0.0142 (3)	
N3	0.78999 (11)	0.37071 (11)	0.48923 (16)	0.0134 (3)	
N4	0.26042 (13)	0.31912 (13)	0.22293 (18)	0.0214 (3)	
H1	0.257 (2)	0.3836 (8)	0.242 (3)	0.038 (7)*	
H2	0.277 (2)	0.2825 (17)	0.3059 (19)	0.041 (7)*	
C3	0.92801 (14)	0.44919 (13)	0.67384 (18)	0.0142 (3)	
C4	1.00186 (13)	0.39722 (13)	0.61098 (18)	0.0127 (3)	
C5	0.96720 (13)	0.32902 (12)	0.48733 (18)	0.0137 (3)	
C6	1.04567 (15)	0.26894 (15)	0.4208 (2)	0.0208 (4)	
H6A	1.0069	0.2343	0.3286	0.031*	
H6B	1.0811	0.2188	0.4953	0.031*	
H6C	1.1006	0.3141	0.3946	0.031*	
C7	0.81235 (14)	0.43424 (13)	0.61656 (19)	0.0149 (3)	
C8	0.68056 (13)	0.35427 (13)	0.40510 (19)	0.0145 (3)	
C9	0.65207 (15)	0.25831 (14)	0.3480 (2)	0.0221 (4)	
H9	0.7026	0.2042	0.3701	0.027*	
C10	0.54950 (15)	0.24169 (14)	0.2583 (2)	0.0219 (4)	
H10	0.5293	0.1762	0.2187	0.026*	
C11	0.47709 (13)	0.32119 (14)	0.22727 (19)	0.0161 (3)	
C12	0.50573 (15)	0.41714 (14)	0.2838 (2)	0.0226 (4)	
H12	0.4552	0.4711	0.2611	0.027*	
C13	0.60796 (15)	0.43436 (14)	0.3732 (2)	0.0202 (4)	
H13	0.6282	0.5000	0.4122	0.024*	

### Atomic displacement parameters (Å^2^)

**Table:**

	*U*^11^	*U*^22^	*U*^33^	*U*^12^	*U*^13^	*U*^23^
S1	0.0092 (2)	0.0149 (2)	0.0165 (2)	−0.00054 (15)	0.00014 (15)	−0.00010 (15)
S2	0.0100 (2)	0.0256 (3)	0.0150 (2)	0.00070 (16)	−0.00154 (15)	−0.00466 (16)
O1	0.0139 (6)	0.0306 (8)	0.0217 (6)	0.0011 (5)	0.0038 (5)	−0.0071 (5)
O2	0.0162 (7)	0.0324 (8)	0.0384 (8)	0.0005 (6)	−0.0042 (6)	−0.0187 (7)
O3	0.0172 (7)	0.0496 (10)	0.0211 (7)	0.0025 (6)	−0.0020 (5)	0.0096 (6)
C1	0.0185 (9)	0.0209 (9)	0.0190 (8)	−0.0030 (7)	−0.0015 (7)	−0.0042 (7)
C2	0.0165 (8)	0.0140 (8)	0.0150 (8)	−0.0001 (7)	0.0015 (6)	0.0000 (6)
N1	0.0130 (7)	0.0175 (7)	0.0164 (7)	−0.0013 (6)	0.0006 (5)	−0.0028 (6)
N2	0.0118 (7)	0.0138 (7)	0.0163 (7)	0.0014 (5)	0.0018 (5)	−0.0004 (5)
N3	0.0086 (7)	0.0146 (7)	0.0162 (7)	0.0002 (5)	0.0006 (5)	−0.0013 (5)
N4	0.0143 (7)	0.0297 (9)	0.0197 (8)	−0.0001 (7)	0.0027 (6)	−0.0038 (7)
C3	0.0146 (8)	0.0136 (8)	0.0136 (7)	0.0000 (6)	0.0012 (6)	0.0012 (6)
C4	0.0102 (8)	0.0137 (8)	0.0132 (7)	−0.0007 (6)	0.0002 (6)	0.0028 (6)
C5	0.0117 (8)	0.0132 (8)	0.0158 (7)	0.0007 (6)	0.0017 (6)	0.0016 (6)
C6	0.0137 (8)	0.0237 (9)	0.0234 (9)	0.0030 (7)	0.0007 (7)	−0.0072 (7)
C7	0.0122 (8)	0.0164 (8)	0.0155 (8)	0.0005 (6)	0.0015 (6)	0.0001 (6)
C8	0.0090 (8)	0.0173 (8)	0.0158 (8)	−0.0006 (6)	−0.0008 (6)	0.0009 (6)
C9	0.0165 (9)	0.0159 (9)	0.0299 (10)	0.0021 (7)	−0.0042 (7)	0.0008 (7)
C10	0.0167 (9)	0.0161 (9)	0.0293 (10)	−0.0017 (7)	−0.0032 (7)	−0.0036 (7)
C11	0.0096 (8)	0.0217 (9)	0.0154 (7)	−0.0001 (7)	−0.0011 (6)	−0.0003 (7)
C12	0.0161 (9)	0.0197 (9)	0.0289 (10)	0.0060 (7)	−0.0020 (8)	−0.0030 (7)
C13	0.0151 (9)	0.0152 (9)	0.0275 (9)	0.0011 (7)	−0.0021 (7)	−0.0047 (7)

### Geometric parameters (Å, °)

**Table:**

S1—C4	1.7142 (17)	N4—H2	0.875 (10)
S1—C2	1.7502 (17)	C3—C4	1.371 (2)
S2—O3	1.4288 (15)	C3—C7	1.453 (2)
S2—O2	1.4326 (15)	C4—C5	1.425 (2)
S2—N4	1.6105 (16)	C5—C6	1.489 (2)
S2—C11	1.7674 (17)	C6—H6A	0.9800
O1—C7	1.231 (2)	C6—H6B	0.9800
C1—C2	1.488 (2)	C6—H6C	0.9800
C1—H1A	0.9800	C8—C9	1.387 (3)
C1—H1B	0.9800	C8—C13	1.390 (2)
C1—H1C	0.9800	C9—C10	1.388 (3)
C2—N1	1.306 (2)	C9—H9	0.9500
N1—C3	1.376 (2)	C10—C11	1.382 (3)
N2—C5	1.300 (2)	C10—H10	0.9500
N2—N3	1.3746 (19)	C11—C12	1.386 (3)
N3—C7	1.398 (2)	C12—C13	1.385 (3)
N3—C8	1.441 (2)	C12—H12	0.9500
N4—H1	0.872 (10)	C13—H13	0.9500
			
C4—S1—C2	88.46 (8)	N2—C5—C4	120.34 (15)
O3—S2—O2	118.12 (9)	N2—C5—C6	117.64 (15)
O3—S2—N4	106.80 (9)	C4—C5—C6	122.01 (15)
O2—S2—N4	107.68 (9)	C5—C6—H6A	109.5
O3—S2—C11	108.86 (8)	C5—C6—H6B	109.5
O2—S2—C11	107.57 (8)	H6A—C6—H6B	109.5
N4—S2—C11	107.36 (8)	C5—C6—H6C	109.5
C2—C1—H1A	109.5	H6A—C6—H6C	109.5
C2—C1—H1B	109.5	H6B—C6—H6C	109.5
H1A—C1—H1B	109.5	O1—C7—N3	121.93 (15)
C2—C1—H1C	109.5	O1—C7—C3	125.48 (16)
H1A—C1—H1C	109.5	N3—C7—C3	112.59 (14)
H1B—C1—H1C	109.5	C9—C8—C13	120.93 (16)
N1—C2—C1	125.00 (16)	C9—C8—N3	118.39 (15)
N1—C2—S1	115.69 (13)	C13—C8—N3	120.53 (15)
C1—C2—S1	119.30 (13)	C8—C9—C10	119.70 (17)
C2—N1—C3	109.40 (14)	C8—C9—H9	120.2
C5—N2—N3	118.94 (14)	C10—C9—H9	120.2
N2—N3—C7	126.57 (14)	C11—C10—C9	119.39 (17)
N2—N3—C8	111.88 (13)	C11—C10—H10	120.3
C7—N3—C8	121.55 (14)	C9—C10—H10	120.3
S2—N4—H1	109.6 (17)	C10—C11—C12	120.90 (16)
S2—N4—H2	110.8 (18)	C10—C11—S2	119.45 (14)
H1—N4—H2	113 (2)	C12—C11—S2	119.64 (14)
C4—C3—N1	116.28 (15)	C13—C12—C11	120.05 (17)
C4—C3—C7	120.28 (15)	C13—C12—H12	120.0
N1—C3—C7	123.44 (15)	C11—C12—H12	120.0
C3—C4—C5	120.99 (15)	C12—C13—C8	119.02 (17)
C3—C4—S1	110.16 (12)	C12—C13—H13	120.5
C5—C4—S1	128.86 (13)	C8—C13—H13	120.5
			
C4—S1—C2—N1	1.29 (14)	C4—C3—C7—O1	175.26 (17)
C4—S1—C2—C1	−177.20 (15)	N1—C3—C7—O1	−4.5 (3)
C1—C2—N1—C3	177.37 (16)	C4—C3—C7—N3	−4.9 (2)
S1—C2—N1—C3	−1.03 (19)	N1—C3—C7—N3	175.35 (15)
C5—N2—N3—C7	−3.4 (2)	N2—N3—C8—C9	37.7 (2)
C5—N2—N3—C8	176.49 (15)	C7—N3—C8—C9	−142.50 (17)
C2—N1—C3—C4	0.1 (2)	N2—N3—C8—C13	−137.94 (16)
C2—N1—C3—C7	179.88 (16)	C7—N3—C8—C13	41.9 (2)
N1—C3—C4—C5	−179.24 (15)	C13—C8—C9—C10	−0.3 (3)
C7—C3—C4—C5	1.0 (2)	N3—C8—C9—C10	−175.91 (17)
N1—C3—C4—S1	0.84 (19)	C8—C9—C10—C11	0.0 (3)
C7—C3—C4—S1	−178.93 (13)	C9—C10—C11—C12	0.3 (3)
C2—S1—C4—C3	−1.12 (13)	C9—C10—C11—S2	−179.11 (15)
C2—S1—C4—C5	178.97 (16)	O3—S2—C11—C10	−129.82 (16)
N3—N2—C5—C4	−1.4 (2)	O2—S2—C11—C10	−0.72 (18)
N3—N2—C5—C6	178.80 (15)	N4—S2—C11—C10	114.92 (16)
C3—C4—C5—N2	2.5 (2)	O3—S2—C11—C12	50.76 (17)
S1—C4—C5—N2	−177.63 (13)	O2—S2—C11—C12	179.85 (15)
C3—C4—C5—C6	−177.76 (16)	N4—S2—C11—C12	−64.51 (17)
S1—C4—C5—C6	2.1 (3)	C10—C11—C12—C13	−0.3 (3)
N2—N3—C7—O1	−173.80 (15)	S2—C11—C12—C13	179.15 (15)
C8—N3—C7—O1	6.4 (3)	C11—C12—C13—C8	−0.1 (3)
N2—N3—C7—C3	6.3 (2)	C9—C8—C13—C12	0.4 (3)
C8—N3—C7—C3	−173.48 (14)	N3—C8—C13—C12	175.85 (16)

### Hydrogen-bond geometry (Å, °)

**Table:**

*D*—H···*A*	*D*—H	H···*A*	*D*···*A*	*D*—H···*A*
N4—H1···O1^i^	0.87 (1)	2.06 (1)	2.922 (2)	169 (2)
N4—H2···O2^ii^	0.88 (1)	2.38 (2)	3.090 (2)	139 (2)

Symmetry codes: (i) −*x*+1, −*y*+1, −*z*+1; (ii) *x*, −*y*+1/2, *z*+1/2.
